# Supplementary figures and images for: Dynamic topic modeling of twitter data during the COVID-19 pandemic
Source: PLoS One. 2022 May 27;17(5):e0268669. doi: 10.1371/journal.pone.0268669 (PMC9140268; doi:10.1371/journal.pone.0268669)

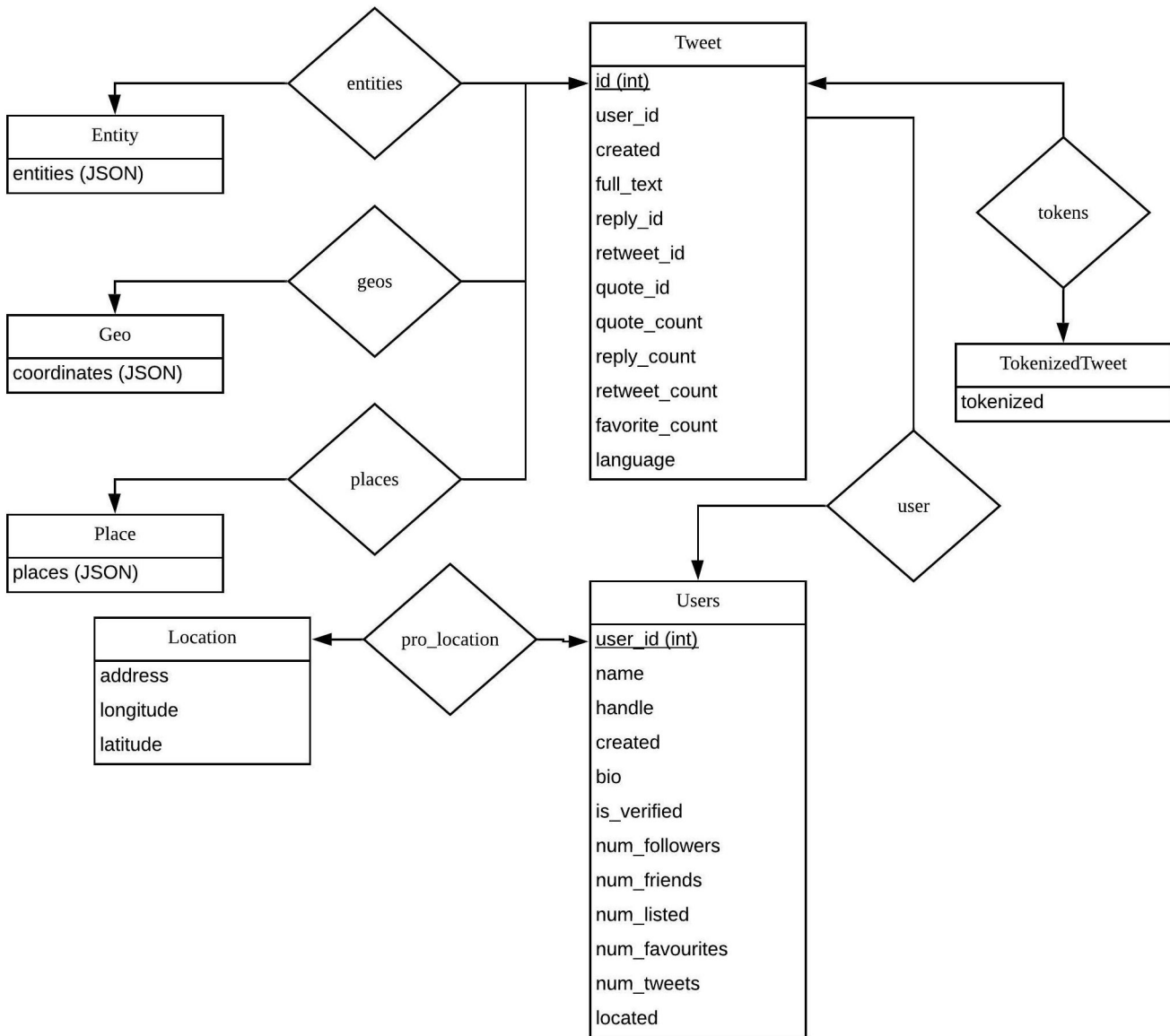

Supplement: S1 Appendix — (PDF) [file pone.0268669.s001.pdf]
