# Supplementary material for: Dynamic topic modeling of twitter data during the COVID-19 pandemic
Source: PLoS One. 2022 May 27;17(5):e0268669. doi: 10.1371/journal.pone.0268669 (PMC9140268; doi:10.1371/journal.pone.0268669)
Supplement: S2 Appendix — (DOCX) [file pone.0268669.s002.docx]

# A. Appendix

| **Day** | **Topic** | **Words** | **Tweet** |
| --- | --- | --- | --- |
| 3 | 46 | ['like', 'time', 'need', 'know', 'help', 'day', 'world', 'work', 'going', 'right'] | A virtual influencer is helping to drive donations in WHO's new COVID-19 campaign https://t.co/tWdBRxSjDv https://t.co/J65xNzareA |
| 3 | 17 | ['mask', 'face_mask', 'help', 'ppe', 'wear',  'worker', 'hospital', 'nurse', 'need', 'wear_mask'] | Thank you from the Manila Protective Gear Sewing Club!! https://t.co/rihggYi4Jp |
| 3 | 21 | ['time', 'support', 'help', 'news', 'need', 'new', 'crisis', 'case', 'community', 'thank'] | Apple highlights apps to help families manage autism amid the coronavirus - CNET https://t.co/UzeWZnQB1D |
| 3 | 52 | ['case', 'county', 'death', 'new', 'state', 'breaking', 'hospital', 'reported', 'trump', 'news'] | A 13th employee at the Cook County Circuit Court clerk’s oﬃce has tested positive for COVID-19. https://t.co/yNP7T6HKHa |
| 3 | 35 | ['staﬀ', 'supply', 'ppe', 'fight', 'medical',  'hospital', 'help', 'testing', 'need', 'country'] | When an IAS oﬃcer wears a full PPE kit and doctors wear normal clothes and mask. I don't think Modi's promise is working here  #uglyindianbureaucracy  @PMOIndia @narendramodi @drharshvardhan @DrHarjitBhatti @drpankajsolanki @UnitedRda @FordaIndia @RajCMO @CMODelhi https://t.co/UqojﬀI5oF |
| 4 | 46 | ['like', 'time', 'need', 'know', 'world', 'think', 'going', 'day', 'help', 'right'] | @narendramodi @dm_ghaziabad @CMOﬃceUP dear sir kindly spare some time to discuss the serious matter regarding pre paid electricity meter and maintenance charges in River Heights Ph 2 Raj Nagar Ext.,Ghaziabad as the builder is enjoying all the liberty against law.kindly act fast |
| 4 | 52 | ['case', 'county', 'death', 'state', 'new', 'new_york', 'breaking', 'reported', 'update', 'total'] | Medical personnel transfer bodies into and out of a refrigerated truck placed outside #Brooklyn Hospital Center in New York City #NYC Wednesday in order to deal with a spike in #COVID19 deaths  #NewYork has confirmed 102,870 cases and at least 2,935 deaths https://t.co/jCCZIa3Okq |
| 4 | 35 | ['supply', 'staﬀ', 'ppe', 'medical', 'fight', 'equipment', 'hospital', 'help', 'government', 'passenger'] | @jensstoltenberg Mr.Stoltenberg, #Turkey is sending everyday #illegal #migrants with #COVID2019 to the Greek Islands. What kind of solidarity is this? Why #NATO didn't stop it?  #europeanborderguard #greece_under_attack |
| 4 | 21 | ['support', 'time', 'help', 'news', 'need', 'new', 'community', 'crisis', 'thank', 'excellent'] | DOH says medical team from China will stay in the country until April 19 |
| 4 | 17 | ['mask', 'face_mask', 'wear_mask', 'wear', 'ppe',  'help', 'nurse', 'hospital', 'worker', 'patient'] | @ME31017974 @Romeites @KKMPutrajaya wear mask 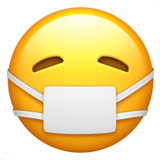. Mask is important so that asymptomatic cases don’😷t continue to spread C19. See video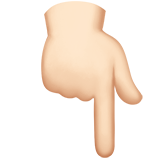.I will be spreading this news internationally but for the time being, since Msia is not receiving anyone frm outside, this is the time we can contain C19 frm inside #FightMrCovid https://t.co/g1j4TToGk5 |
| 5 | 46 | ['like', 'time', 'need', 'know', 'world', 'think', 'going', 'day', 'life', 'right'] | What if the entire world shuts down completely until every last infected person has recovered and the virus is extinct before quarantine ends? And what if you’re that last guy, and the world is tapping it’s foot... |
| 5 | 35 | ['supply', 'staﬀ', 'ppe', 'equipment', 'medical', 'fight', 'hospital', 'player', 'passenger', 'ventilator'] | #COVID19 Govt is giving liberty in paying EMIs, where as #landcraft builder imposing additional financial burden by starting deduction of maintenance charge from pre paid electric meter @dm_ghaziabad @gdagzb @myogioﬃce @JansunwaiAbhiyn |
| 5 | 17 | ['mask', 'face_mask', 'ppe', 'nurse', 'worker',  'hospital', 'patient', 'wear_mask', 'wear', 'help'] | In our #COVID19 blog post, we shared this 45 second "How to" video from the #SurgeonGeneral on creating your #facemasks at home. We'll be doing this to help ensure we aren't inadvertantly spreading the virus even though we are asymptomatic:  https://t.co/GVOA15MQ5x  #cdc https://t.co/3TwWWMr8C0 |
| 5 | 52 | ['case', 'death', 'county', 'new', 'state',  'new_york', 'breaking', 'total', 'update', 'reported'] | 'Now is not the time to discuss pay rise for nurses', says health secretary Matt Hancock.  You still clapping for the NHS but voting Tory? Mad. |
| 6 | 46 | ['like', 'time', 'need', 'know', 'world', 'think', 'going', 'day', 'life', 'right'] | “No new fracking bans without scientific research” going well, @GavinNewsom. Way to be a climate leader! https://t.co/4dybYJ7FTo |
| 6 | 21 | ['support', 'help', 'community', 'time', 'crisis',  'thank', 'excellent', 'need', 'news', 'new'] | Korea is managing the COVID-19 crisis by emphasizing transparency and open communication, public-private partnerships, evidence-based deployment of public health measures, and innovative use of technology and data.&amp;nbsp; https://t.co/4epOdXkh2k via @WB_AsiaPacific |
| 6 | 35 | ['supply', 'staﬀ', 'equipment', 'ppe', 'medical',  'fight', 'hospital', 'player', 'passenger', 'worker'] | Working from home- the desperation 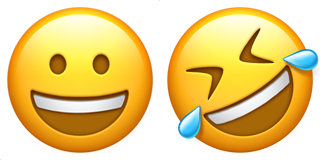 😀 🤣  #covid19 #coronavirus #humour https://t.co/q2hSnyh2Vp |
| 6 | 17 | ['mask', 'nurse', 'ppe', 'worker', 'hospital',  'face_mask', 'patient', 'medical', 'wear', 'help'] | Q&amp;A on coronaviruses (COVID-19)  Most common symptoms of #COVID19 are fever, tiredness, &amp; dry cough. Some: aches &amp; pains, nasal congestion, runny nose, sore throat or diarrhea.  People with fever, cough &amp; diﬃculty breathing should seek medical attention. https://t.co/hOdLwM6qbV |
| 6 | 52 | ['case', 'death', 'new_york', 'new', 'state',  'county', 'breaking', 'update', 'total', 'reported'] | VDH Daily Update: 2878 Coronavirus Cases in Va. with 54 Deaths https://t.co/RiVf8Y857l |
| 7 | 46 | ['like', 'time', 'need', 'know', 'world', 'day', 'think', 'going', 'life', 'right'] | This ain’t it... IDK what the city is doing but they need to reapply that money into the youth another way if they are not going to provide jobs. |
| 7 | 17 | ['mask', 'nurse', 'worker', 'ppe', 'hospital',  'patient', 'face_mask', 'medical', 'health', 'help'] | Nurses are not getting the protections they desperately need to fight #COVID19. @NationalNurses is demanding Congress act now. Add your name: https://t.co/YTc3RGD6Xw |
| 7 | 52 | ['case', 'death', 'new', 'new_york', 'state',  'county', 'breaking', 'update', 'total', 'city'] | Nearly 1 in 6 people who have died from the new coronavirus in New York state lived in a nursing home. #NY1Health https://t.co/lSWKiGRIxf |
| 7 | 21 | ['support', 'community', 'thank', 'help', 'health',  'crisis', 'time', 'world', 'excellent', 'need'] | 2/ for @TheContentMine openVirus we need  volunteers who know how to index abstracts and theses using SOLR. We have a great team who can retrieve and a great infrastructure.  Somewhere in the existing scholarly literature are solutions to tackling the pandemic. |
| 7 | 35 | ['supply', 'staﬀ', 'equipment', 'medical', 'ppe',  'fight', 'player', 'worker', 'hospital', 'company'] | Coronavirus Impact: Jackson Memorial Hospital CEO Carlos Migoya Questioned Over ‘Furloughs, Pay Cuts, Not Providing Protective Equipment’ https:// t.co/9ks4JVkAoh |
| 8 | 21 | ['support', 'community', 'thank', 'help', 'health',  'crisis', 'time', 'response', 'world', 'need'] | Proud to have served, and holding vigil for all my brothers and sisters on the front lines in healthcare. #HealthcareHeroes #COVID19 |
| 8 | 46 | ['like', 'time', 'need', 'know', 'world', 'day', 'think', 'going', 'life', 'right'] | My granny is 92 today. Here she is, celebrating away to herself while she stays isolated. Can you fathom the joy? 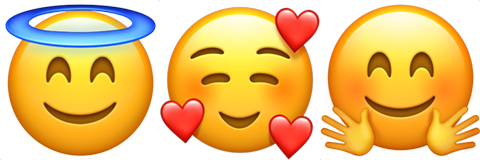https://t.co/CIzw1FrJlP |
| 8 | 35 | ['supply', 'staﬀ', 'equipment', 'medical', 'worker',  'ppe', 'player', 'fight', 'hospital', 'company'] | Hotline service to begin for foreigners in Japan for virus inquiries https://t.co/iXtUdvv4Yi |
| 8 | 52 | ['case', 'death', 'new', 'state', 'new_york',  'county', 'update', 'total', 'breaking', 'city'] | My daily NY COVID-19 tracker:  149,316 confirmed cases  779 deaths |
| 8 | 17 | ['mask', 'worker', 'nurse', 'ppe', 'hospital',  'patient', 'face_mask', 'medical', 'health', 'help'] | #Researchers at @NotreDame have developed micro/nanofluidic devices for isolating cellular material such as #vesicles and #exosomes during #liquidbiopsies. They turn out to be the same size as the #coronavirus. - @NDCBE https://t.co/v3H4U2uu1b |


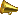

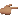


| 9 | 46 | ['like', 'time', 'need', 'know', 'world', 'day', 'going', 'think', 'life', 'good'] | Best estimates of #R0 for #coronavirus seem to be 2-4. and yet it feels like it spreads very easily. So I can’t even imagine what it would feel like if we were in an outbreak of something with a much higher R0 and we didn’t have a vaccine, like #measles. |
| --- | --- | --- | --- |
| 9 | 21 | ['support', 'community', 'thank', 'help', 'health',  'crisis', 'response', 'time', 'need', 'team'] | No matter what challenges we face, we’re always #VegasStrong. @UNLVathletics is going to make and donate 3,000 cloth masks to @UMCSN to respond to the #COVID pandemic—proud to see them stepping up and helping our community in need. https://t.co/yC3uA66eR8 |
| 9 | 52 | ['case', 'death', 'new', 'state', 'new_york',  'county', 'update', 'total', 'breaking', 'city'] | Tasmania's north-west coast residents preparing for 'a whole new world' as coronavirus lockdown looms - ABC News https://t.co/MOqjprpB6U |
| 9 | 17 | ['mask', 'worker', 'nurse', 'ppe', 'hospital', 'patient', 'medical', 'face_mask', 'health', 'protect'] | The @CDCgov recently recommended anyone over 2-years-old should wear a mask outside. Dr. Julia Sammons, Medical Director of our Department of #InfectionPrevention &amp; Control, spoke with @WhatToExpect about how to protect children under 2. #COVID19 https://t.co/IGBjmQRPn9 |
| 9 | 35 | ['supply', 'staﬀ', 'equipment', 'worker', 'ppe',  'medical', 'company', 'player', 'hospital', 'fight'] | As demand spikes, meds used alongside ventilators are in short supply. Manufacturers must ramp up drug production even more, says 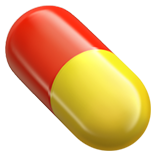drug shortage expert @foxerinr. All #COVID19 patients deserve access to #MedsWeCanTrust: @edsilverman https://t.co/gSJ7jl6z2Q |
| 10 | 46 | ['like', 'time', 'need', 'know', 'world', 'day', 'good', 'going', 'think', 'life'] | A Communal Virus and Our Collective Irrationality - India Gone Viral https://t.co/wgcyb8cOJU |
| 10 | 52 | ['case', 'death', 'new', 'state', 'new_york',  'update', 'county', 'total', 'city', 'breaking'] | New York sees record 1-day rise in COVID-19 deaths  https://t.co/RUq1Vs8oJH |
| 10 | 21 | ['support', 'community', 'thank', 'help', 'health',  'crisis', 'response', 'time', 'team', 'need'] | @ASlavitt @ScottGottliebMD @NIHDirector @NYGovCuomo @GovNedLamont @GovMurphy  are you watching #maddow covering #nursinghome #longtermcare #covid19 outbreaks nationwide? What can we implement as a national plan for this? #elderly #vulnerable #highriskcovid #coronavirus  @maddow |
| 10 | 35 | ['supply', 'staﬀ', 'equipment', 'ppe', 'worker',  'company', 'medical', 'player', 'hospital', 'fight'] | A #Canadian cargo company says international air carriers are #pricegouging the cost to ship products. It says there is minimal cargo space available due to the #COVIDー19 pandemic, and prices are changing daily. @JGaidolaCHCH has the details  WATCH: https://t.co/9jprmkdutt https://t.co/ZmECk2sRma |
| 10 | 17 | ['mask', 'worker', 'nurse', 'ppe', 'hospital', 'patient', 'medical', 'face_mask', 'health', 'protect'] | How Did the U.S. End Up with Nurses Wearing Garbage Bags? https://t.co/ibg2XtAUYE |
| 11 | 46 | ['like', 'time', 'need', 'know', 'world', 'day', 'think', 'good', 'going', 'life'] | Italian Prime Minister Giuseppe Conte floated the idea of all of Europe doing “things on their own” due to the European Union’s poor response to Italy’s tragic experience with the Chinese coronavirus. https://t.co/e1fv4z2OyR |
| 11 | 17 | ['mask', 'worker', 'nurse', 'ppe', 'hospital', 'patient', 'medical', 'face_mask', 'frontline', 'health'] | To the health care and essential workers putting themselves on the line during this pandemic -- we owe you a massive debt of gratitude.  That’s why this week, I proposed the COVID-19 Heroes Fund to give our frontline workers the compensation they deserve. https://t.co/2JlnH6fiyp |
| 11 | 52 | ['case', 'death', 'new', 'state', 'new_york',  'update', 'total', 'county', 'city', 'breaking'] | #COVID19 update as of 6:00 pm: #Austin now has 744 confirmed cases, up 54 from yesterday. We remain at 9 deaths &amp; have had 133 people recover. We are starting to see an increase in cases in the 40-59 age range, &amp; while cases are rising, they’ve been at a steady rate (1/7) https://t.co/ DmtN2ORxyx |
| 11 | 21 | ['support', 'community', 'thank', 'help', 'health',  'crisis', 'response', 'team', 'time', 'need'] | leading Muslim charities from across the UK have united to pool their resources, skills and expertise ensuring that they can eﬃciently provide support to where it’s most needed  support and share if you can pls  Campaign for National Solidarity COVID-19 https://t.co/R3JTXkIprF |
| 11 | 35 | ['supply', 'staﬀ', 'equipment', 'ppe', 'worker',  'company', 'medical', 'player', 'hospital', 'fight'] | 📣 Sign our petition calling on food delivery companies to cut their commissions! #CommunityOverCommission  👉 https://t.co/GMszvxuBPe |
| 12 | 46 | ['like', 'time', 'need', 'know', 'world', 'day', 'life', 'think', 'going', 'good'] | @HusainHacker Happy birthday! May God bless u with some jari butti so that tum safe raho Corona virus se 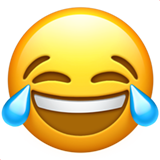 |
| 12 | 52 | ['case', 'death', 'new', 'state', 'new_york', 'total', 'update', 'county', 'city', 'breaking'] | Green Lake County Public Health said the person was hospitalized and is in stable condition. https://t.co/jOSl89vyMx |
| 12 | 38 | ['easter', 'god', 'jesus', 'church', 'lord', 'pray', 'christian', 'wash', 'prayer', 'holy'] | HAVE A BLESSED EASTER!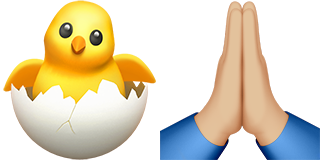 🐣 🙏  Rejoice in the risen Christ!  Rejoice by faith!  Rejoice when we are still in our night, ( #covid19 )  for Jesus is the Light Who is sure to dawn.  (2 Pt 1:19; cf Rv 22:16). Alleluia forever! https://t.co/Yr9XIQUhoN |
| 12 | 21 | ['support', 'community', 'thank', 'help', 'health',  'crisis', 'response', 'team', 'excellent', 'time'] | BP Oman has chartered a flight to bring back students and also dependents of Omanis from the UK in cooperation with @MofaOman, @OmanEmbassyUK and @omanair as part of our response to Covid-19.  @BP_Oman https://t.co/o6JrZMXvzz |
| 12 | 17 | ['mask', 'worker', 'nurse', 'ppe', 'hospital', 'patient', 'medical', 'face_mask', 'frontline', 'protect'] | "It was the police who sloughed oﬀ social-distancing: physically handling him, cuﬃng him without wearing protective gear as unworn masks dangled from their belts" https://t.co/firTL6IJME |
| 13 | 46 | ['time', 'like', 'need', 'know', 'world', 'day', 'life', 'think', 'going', 'good'] | Coronavirus brings out the worst haircuts in all of us. |
| 13 | 17 | ['mask', 'worker', 'nurse', 'ppe', 'hospital', 'patient', 'medical', 'frontline', 'face_mask', 'healthcare_worker'] | A major California labor union that claimed to have discovered a stockpile of 39 million masks for health care workers fighting the coronavirus was duped in an elaborate scam uncovered by FBI investigators.  https://t.co/CP1zegIOQl |
| 13 | 52 | ['case', 'death', 'new', 'state', 'new_york', 'total', 'update', 'county', 'city', 'reported'] | The new cases brings the total to 17 in the county https://t.co/7OYoHs34Kq |
| 13 | 21 | ['support', 'community', 'thank', 'help', 'crisis',  'health', 'response', 'team', 'excellent', 'time'] | Here’s our roundup of the Top Ten resources about coronavirus. Atrium Health experts weighed in to separate fact from fiction.  Keep this link handy as you prepare for the week ahead. https://t.co/m5JTofSY7S #AtriumHealthProud |
| 13 | 35 | ['supply', 'staﬀ', 'ppe', 'company', 'equipment',  'worker', 'player', 'medical', 'employee', 'testing'] | Farm workers cannot carry on with harvesting and planting, with truckers and air freight capacity having ground to a halt due to #COVID19 crisis. #FoodAndRightsNow  https://t.co/GBvQg7WRXP |
